# Supplementary material for: Social distancing beliefs and human mobility: Evidence from Twitter
Source: PLoS One. 2021 Mar 3;16(3):e0246949. doi: 10.1371/journal.pone.0246949 (PMC7928458; doi:10.1371/journal.pone.0246949)
Supplement: S1 File — (PDF) [file pone.0246949.s001.pdf]

Appendix A

Fig A1. Twitter - Number of Social Distancing tweets per day.

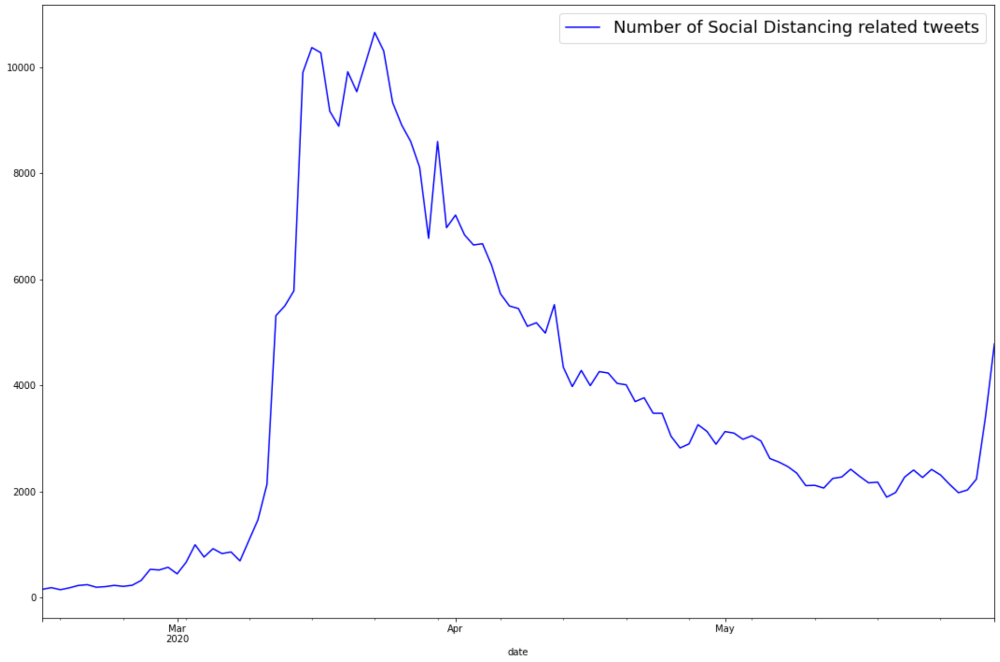

Notes: This Figure presents the number of tweets per day encouraging social distancing behaviors.

Appendix B: Additional Statistics and Robustness Checks

Table B1. Means, standard deviations and correlations of independent variables

Variable	mean	sd								
TwitterSD$_{t-1}$	1.048	1.48	1							
CovidCases$_{t-1}$	4.857	7.473	0.171	1						
Stay-at-home Orders	0.479	0.500	0.095	0.418	1					
School Closures	0.701	0.458	0.251	0.412	0.612	1				
Gathering Restrictions	0.646	0.478	0.260	0.383	0.644	0.793	1			
Business Closures	0.372	0.483	0.109	0.394	0.673	0.482	0.493	1		
Precipitations	29.536	56.438	0.032	0.068	0.075	0.094	0.093	0.055	1	
Temperature	155.722	80.068	-0.010	0.164	0.294	0.432	0.370	0.166	0.0826	1

Table B2. Checking for multicollinearity

Variable	Variance Inflation Factor
TwitterSD$_{t-1}$	3.48
COVID_Cases$_{t-1}$	2.23
Stay-at-home Orders	4.59
School Closures	13.89
Gathering Restrictions	5.33
Business Closures	4.62
Precipitations	1.24
Temperature	5.05
Average for Time	2.66
Average for States	2.24
**Mean for all variables**	**2.65**

Table B3. Impact of Twitter indices of social distancing weighted by likes, retweets and replies on mobility

VARIABLES	(1)<br>Workplaces	(2)<br>Groceries and pharmacies	(3)<br>Retail and recreation	(4)<br>Transit stations	(5)<br>Parks
z_TwitterSD$_{t-1}$	-1.220***	-0.347*	-0.471**	-1.546***	-1.684***
	(0.169)	(0.190)	(0.225)	(0.297)	(0.558)
R-squared	0.981	0.920	0.967	0.945	0.824
z_TwitterSD_Likes$_{t-1}$	-0.841***	-0.185	-0.321**	-1.094***	-1.285***
	(0.103)	(0.174)	(0.152)	(0.214)	(0.478)
R-squared	0.981	0.920	0.967	0.945	0.824
z_TwitterSD_Retweets$_{t-1}$	-0.767***	-0.187	-0.328**	-1.053***	-1.404***
	(0.0867)	(0.168)	(0.136)	(0.199)	(0.405)
R-squared	0.980	0.920	0.967	0.945	0.824
z_TwitterSD_Replies$_{t-1}$	-0.753***	-0.209	-0.369***	-1.068***	-1.382***
	(0.0764)	(0.152)	(0.136)	(0.190)	(0.391)
R-squared	0.980	0.920	0.967	0.945	0.824
Observations	5,194	5,194	5,194	5,194	5,183
State FE	Yes	Yes	Yes	Yes	Yes
Division*Time FE	Yes	Yes	Yes	Yes	Yes
# Variables	1,001	1,001	1,001	1,001	1,001

Note: All models are OLS regressions with state and division*time FE. All controls from the baseline regression are included but not reported for ease in reading. Twitter indices are standardized and lagged by one day. State-level clustered robust standard errors in parentheses with *** p<0.01, ** p<0.05, * p<0.1.

Table B4. Baseline Results using [25]'s and [26]'s models

	(1)	(2)	(3)	(4)	(5)	(6)
VARIABLES	Residential	Workplaces	Groceries and pharmacies	Retail and recreation	Transit stations	Parks
			Using Newey and West's standard errors			
TwitterSD_{t-1}	0.295***	-0.827***	-0.235	-0.319	-1.047***	-1.141
	(0.0858)	(0.197)	(0.164)	(0.292)	(0.346)	(0.790)
CovidCases_{t-1}	0.0927***	-0.150***	-0.127***	-0.227***	-0.256***	-0.598***
	(0.00905)	(0.0187)	(0.0235)	(0.0284)	(0.0371)	(0.103)
			Using Driscoll and Kraay's standard errors			
TwitterSD_{t-1}	0.295**	-0.827***	-0.235	-0.319	-1.047*	-1.141
	(0.125)	(0.277)	(0.244)	(0.413)	(0.552)	(1.229)
CovidCases_{t-1}	0.0927***	-0.150***	-0.127***	-0.227***	-0.256***	-0.598***
	(0.0191)	(0.0322)	(0.0355)	(0.0432)	(0.0740)	(0.115)
Observations	5,194	5,194	5,194	5,194	5,194	5,183
Division*Time FE	Yes	Yes	Yes	Yes	Yes	Yes

Note: [25] standard errors are computed using an OLS model. [26] standard errors are computed using a State fixed-effect model. All models have division*time FE dummies. Controls are included but their coefficients are not reported for ease in reading. Standard errors in parentheses with *** p<0.01, ** p<0.05, * p<0.1.

Table B5. Baseline Results using alternative lags and first differences

	(1)	(2)	(3)	(4)	(5)	(6)
VARIABLES	Residential	Workplaces	Groceries and pharmacies	Retail and recreation	Transit stations	Parks
			Using 2 lags for TwitterSD and CovidCases			
$TwitterSD_{t-1}$	0.0873**	-0.163	-0.241**	-0.168*	-0.169	-0.848
	(0.0425)	(0.113)	(0.110)	(0.0908)	(0.163)	(0.614)
$TwitterSD_{t-2}$	0.0754**	-0.255***	0.0512	0.0311	-0.153	0.115
	(0.0345)	(0.0828)	(0.100)	(0.0936)	(0.114)	(0.643)
$CovidCases_{t-1}$	0.0213***	-0.0226***	-0.0318	-0.0430***	-0.0146	-0.275**
	(0.00606)	(0.00810)	(0.0195)	(0.0151)	(0.0178)	(0.117)
$CovidCases_{t-2}$	0.0252***	-0.0400***	-0.0472**	-0.0474***	-0.0366**	-0.141
	(0.00583)	(0.0122)	(0.0186)	(0.0139)	(0.0166)	(0.117)
Lagged Mobility	0.561***	0.615***	0.513***	0.692***	0.785***	0.349***
	(0.0349)	(0.0245)	(0.0420)	(0.0415)	(0.0287)	(0.0582)
R-squared	0.983	0.988	0.942	0.983	0.979	0.846
			Using first differences for continuous variables			
Δ $TwitterSD_{t-1}$	0.106**	-0.181**	-0.486***	-0.220*	-0.130	-1.050
	(0.0443)	(0.0895)	(0.140)	(0.113)	(0.149)	(1.028)
Δ $CovidCases_{t-1}$	-0.00387	0.0148*	0.00190	0.000778	0.0169	-0.0381
	(0.00510)	(0.00828)	(0.0135)	(0.00933)	(0.0160)	(0.113)
R-squared	0.927	0.937	0.766	0.775	0.641	0.616
Observations	5,194	5,194	5,194	5,194	5,194	5,183
Division*Time FE	Yes	Yes	Yes	Yes	Yes	Yes

Note: All models are OLS models with state and Division*Time fixed effects. In the first part of the table, we use two lags for the Twitter index of social distancing and COVID-19 cases, and one lag for the dependent variable. In the second part of the table, we use the first difference for all continuous variables, including mobility. In the latter case, the dependent variable is the first difference of the considered mobility between t and t-1. Controls are included but their coefficients are not reported for ease in reading. State-level clustered robust standard errors in parentheses with *** p<0.01, ** p<0.05, * p<0.1.
